# Alternative Splicing and Transcriptome Profiling of Experimental Autoimmune Encephalomyelitis Using Genome-Wide Exon Arrays

**DOI:** 10.1371/journal.pone.0007773

**Published:** 2009-11-10

**Authors:** Alan Gillett, Klio Maratou, Chris Fewings, Robert A. Harris, Maja Jagodic, Tim Aitman, Tomas Olsson

**Affiliations:** 1 Department of Clinical Neuroscience, Karolinska Institutet, Neuroimmunology Unit, Center for Molecular Medicine, Karolinska Hospital at Solna, Stockholm, Sweden; 2 Clinical Sciences Centre, Physiological Genomics and Medicine Group, Hammersmith Hospital, London, United Kingdom; Centre de Regulació Genòmica, Spain

## Abstract

**Background:**

Multiple Sclerosis (MS) is a chronic inflammatory disease causing demyelination and nerve loss in the central nervous system. Experimental autoimmune encephalomyelitis (EAE) is an animal model of MS that is widely used to investigate complex pathogenic mechanisms. Transcriptional control through isoform selection and mRNA levels determines pathway activation and ultimately susceptibility to disease.

**Methodology/Principal Findings:**

We have studied the role of alternative splicing and differential expression in lymph node cells from EAE-susceptible Dark Agouti (DA) and EAE-resistant Piebald Virol Glaxo.AV1 (PVG) inbred rat strains using Affymetrix Gene Chip Rat Exon 1.0 ST Arrays. Comparing the two strains, we identified 11 differentially spliced and 206 differentially expressed genes at day 7 post-immunization, as well as 9 differentially spliced and 144 differentially expressed genes upon autoantigen re-stimulation. Functional clustering and pathway analysis implicate genes for glycosylation, lymphocyte activation, potassium channel activity and cellular differentiation in EAE susceptibility.

**Conclusions/Significance:**

Our results demonstrate that alternative splicing occurs during complex disease and may govern EAE susceptibility. Additionally, transcriptome analysis not only identified previously defined EAE pathways regulating the immune system, but also novel mechanisms. Furthermore, several identified genes overlap known quantitative trait loci, providing novel causative candidate targets governing EAE.

## Introduction

Multiple Sclerosis (MS) is a complex chronic inflammatory disease primarily affecting young adults. Patients experience deregulated inflammation in the periphery leading to the generation of autoreactive cells that migrate to the target organ, the central nervous system (CNS) [1]. The triggers and factors determining the underlying pathogenic peripheral immune response are unknown; however, modulation of the immune system and lymphocyte trafficking both serve to perturb disease. Use of general immunosuppressive drugs such as mitoxantrone, depleting antibodies against B-cells or antibodies that prevent CNS infiltration all reduce clinical disease [2], [3], [4]. Despite this, secondary damage to neurons and axons causes a progressive disability with no currently effective therapeutic options [5].

Myelin oligodendrocyte glycoprotein (MOG)-induced experimental autoimmune encephalomyelitis (EAE) is an animal model of MS with similarities in pathogenicity and histopathology [6]. Inbred rat strains differ in their susceptibility to disease, permitting the study of susceptible genetic determinants and pathogenic mechanisms that give insight into human MS. We previously described the disease kinetics of EAE-susceptible Dark Agouti (DA) and major histocompatibility complex (MHC)-identical but EAE-resistant Piebald Virol Glaxo.AV1 (PVG) rats in peripheral lymph nodes and the spinal cord [7]. We recorded differences in T helper (T_H_) cell differentiation and regulation of inflammatory markers in lymph nodes at day 7 post-EAE induction. We also characterized effector functions following re-stimulation with autoantigen. However, our previous work only focused on known disease-associated pathways including T_H_1 [8] and T_H_17 [9].

Many studies suggest EAE susceptibility and disease mechanisms are regulated at the level of transcription. Differential expression of genes related to antigen processing and presentation [10], chemokines, cytokines and apoptosis [11], as well as extracellular matrix, cell adhesion molecules and molecules involved in cell division, death and transcription [12], have all been reported. Several of these pathways have also been identified in MS [13], [14], [15]. Furthermore, alternative splicing of genes may result in altered function important for disease pathogenesis. The MS associated interleukin-7 receptor (*IL-7R*) gene [16] is suspected to be involved in MS etiology through the differential splicing of membrane-bound and soluble forms [17]. However, genome-wide investigation of alternative splicing in MS or EAE has not yet been addressed.

In this study we carried out a well powered study of the EAE-susceptible DA and EAE-resistant PVG strains using Affymetrix GeneChip Rat Exon 1.0 ST Arrays to assess exon- and gene-level expression differences in *ex vivo* and MOG re-stimulated lymph node cells. We identify several genes that are alternatively spliced between the strains and may govern disease-driving pathways. In addition, we examine expression differences identifying novel candidates and pathways that associate with disease induction and effector phase functions. Furthermore, several of the genes overlap known quantitative trait loci (QTLs), providing novel candidate targets controlling EAE susceptibility.

## Materials and Methods

### Ethics Statement

All experiments in this study were approved and performed in accordance with the guidelines from the Swedish National Board for Laboratory Animals and the European Community Council Directive (86/609/EEC) under the ethical permit N332/06 entitled ‘Genetic regulation, pathogenesis and therapy of EAE, an animal model for multiple sclerosis’, which was approved by the North Stockholm Animal Ethics Committee (Stockholms norra djurförsöksetiska nämnd). Rats were tested according to a health-monitoring program at the National Veterinary Institute (Statens Veterinärmedicinska Anstalt, SVA) in Uppsala, Sweden.

### Animals and EAE Induction

Inbred DA rats were originally obtained from the Zentralinstitut für Versuchstierzucht (Hannover, Germany) and MHC-identical PVG rats from Harlan UK Limited (Blackthorn, UK). Animals were bred in the animal facility at Karolinska Hospital (Stockholm, Sweden) in a pathogen-free and climate-controlled environment in polystyrene cages containing aspen wood shavings with free access to standard rodent chow and water with regulated 12-hour light/dark cycles. MOG, amino acids 1-125 from the N terminus, was expressed in *Escherichia coli* and purified to homogeneity by chelate chromatography [18]. The purified protein, dissolved in 6M urea, was dialyzed against phosphate buffered saline (PBS) to obtain a physiological preparation that was stored at −20°C. Female rat's aged 10–12 weeks were anaesthetized with isoflurane (Forene, Abbott Laboratories, Chicago, IL, USA) and injected subcutaneously in the tail base in order to induce EAE with a 200 µl inoculum containing 15 µg MOG in PBS, emulsified 1∶1 with incomplete Freund's adjuvant (Sigma-Aldrich, St. Louis, MO, USA).

### Tissue Collection and Cell Culture

Animals were sacrificed using CO_2_ 7 days post-EAE induction, before debut of clinical disease signs. Draining inguinal lymph nodes were collected and placed in DMEM (Gibco-BRL, Grand Island, NY, USA) enriched with 5% fetal calf serum, 1% L-glutamine, 1% penicillin-streptomycin, 1% pyruvic acid (all from Life Technologies, Paisley, Scotland) and 50 µM 2-Mercaptoethanol (‘complete media’; Gibco-BRL) before being mechanically separated by passage through a mesh screen with the bolus of a syringe. Cells were spun at 300 g, resuspended in complete media and 20×10^6^ cells were plated in 10 cm Nunclon Delta Surface Petri Dishes (Nunc, Roskilde, Denmark). Cultures were stimulated for 24 hrs with 25 µg/ml of the encephalogenic MOG_91–108_ peptide (PPR, Downton, UK). Following stimulation the cultured cells were resuspended, washed with PBS, spun and resuspended in 1 ml of TRIzol (Invitrogen, Carlsband, CA, USA). Cells that were not allocated to cell culture (greater than 12×10^6^) were washed with PBS, spun and resuspended in 1 ml of TRIzol. Samples were snap frozen in liquid nitrogen and stored at −70°C.

### Flow Cytometry

Lymph node cells were washed with cold PBS and resuspended in a further 100 µl of PBS. Cells were stained for 20 min at 4°C with the following antibodies: CD3-APC, CD4-PE, CD8a-PE, and CD45RA-PE:Cy5 (all from BD Biosciences, San Jose, CA, USA). Staining was visualized on a FACS Calibur (BD, Franklin Lakes, NJ, USA) with Cell Quest (version 3.2.1f1, BD) and analyzed using FlowJo (version 8.8; Tree Star Inc., Ashland, Oregon).

### Exon Sequencing

Genomic DNA was isolated using a standard protocol. PCR was performed with primers surrounding differentially spliced exons. The amplified products were run on a 1% agarose (Sigma-Aldrich) electrophoresis gel. Bands were extracted and purified using a QIAquick Gel Extraction Kit (Qiagen, Valencia, CA, USA). Sequencing reactions were done at MWG (Ebersberg, Germany). Alignment was performed in Vector NTI Advance v.10 (Invitrogen).

### Quantitative Real-Time PCR

Lymph node *ex vivo* or *in vitro* stimulated cells were washed with PBS before being resuspended in RLT buffer (Qiagen). RNA was purified using an RNeasy kit (Qiagen) and cDNA subsequently prepared using the iScript kit (Bio-Rad, Hercules, CA, USA). Quantitative real-time PCR (qPCR) was performed using a BioRad iQ5 iCycler Detection System with a two-step PCR protocol (95°C for 10 min followed by 40 cycles of 95°C for 10 sec and 60°C for 30 sec), using SYBR Green (Bio-Rad) as the fluorophore. Relative expression levels, corrected for amplification efficiency, were analyzed using iQ5 v2.0 software (BioRad). The primers used for SYBR Green reactions are listed in Table S1. Mann-Whitney non-parametric tests were performed using GraphPad Prism 5 (GraphPad Software, San Diego, CA, USA).

### RNA Extraction and Array Hybridization

Total RNA was extracted using TRIzol reagent, and further purified and DNase I treated using an RNeasy Mini kit (Qiagen) and RNase-Free DNase Set (Qiagen), according to the manufacturer protocols. RNA concentration and purity was determined through measurement of A260/A280 ratios with a NanoDrop ND-1000 Spectrophotometer (NanoDrop Technologies, Wilmington, DE, USA). Confirmation of RNA quality was assessed using the Agilent 2100 Bioanalyzer (Agilent Technologies, Santa Clara, CA, USA). RNA samples were immediately frozen and stored at −80°C. 1 µg of total RNA was used for each sample. Target labeling, as well as array hybridization, washing and staining were performed as described in the GeneChip Whole Transcript (WT) Sense Target Labeling manual (http://www.affymetrix.com). Arrays were scanned using the GeneChip Scanner 3000 7G (Affymetrix).

### Data Analysis

The microarray data is available in MIAME-compliant (minimum information about a microarray experiment) format at the ArrayExpress database (http://www.ebi.ac.uk/arrayexpress) [19] under accession code E-MEXP-2237. CEL intensity files were produced using GeneChip Operating Software version 1.4 (Affymetrix) and quality tested using the Affymetrix Expression Console. All 16 files were deemed suitable for further study. Probe-level data were normalized using Robust Multi-array Average (RMA) [20]. Detection of alternative splicing events was performed using three parallel approaches: 1) Calculations of Microarray Detection of Alternative Splicing (MiDAS; Alternative Transcript Analysis Methods for Exon Arrays; Affymetrix-White-Paper); 2) Rank Product of Splice Index [21] (100 permutations) were performed in R v2.6.2 with the Bioconductor v2.4 [22] package OneChannelGUI v1.4.5 [23]; 3) An Alternative Splice ANOVA model implemented in Partek Genomics Suite v6.4 (Partek Incorporated, St.Louis, MO, USA) together with a filter to select for probe-sets showing significant alternative splicing score, determined at a 5% False Discovery Rate (FDR) [24], and no differential expression at gene level (transcript p>0.05). Transcripts that were significant from all three approaches were visually inspected in the Partek's Gene View to generate lists of alternative splicing candidates. Detection of differential expression at the gene level was performed in Partek Genomics Suite 6.4 (Partek Incorporated). Data was summarized at the gene level using a One-Step Tukey's Biweight Algorithm, which reduces the effect of outlier probe-sets (Statistical Algorithms Description Document; Affymetrix-White-Paper). An ANOVA model, using strain, condition and batch as co-factors, was used to generate raw p values, while FDR was used for multiple test corrections. Genes with 5% FDR were classified as differentially expressed.

### Functional Association Analysis

The lists of differentially expressed genes were uploaded into The Database for Annotation, Visualization and Integrated Discovery v6 (DAVID; http://david.abcc.ncifcrf.gov/) [25], [26] to determine differentially regulated pathways using the full rat genome as reference background. Data were analyzed in the “Functional Annotation Clustering” tool using the “High” classification stringency setting for Molecular Function (MF) and Biological Process (BP) Gene Ontology (GO) terms. Functional annotation clusters with enrichment scores >1.3 were considered significant, in accordance with DAVID recommendations [26]. Pathway analysis was also performed using non-parametric Gene Set Enrichment Analysis (GSEA), v.2 [27]. GSEA considers and ranks all genes in an experiment (not only those above an arbitrary cutoff). It then tests if the list of ranked genes is significantly enriched for pre-defined groups of genes, or “gene sets”. For these analysis 1191 gene sets were used, which comprise the entire C5/GO gene sets collection of MSigDB version 2.5 [27], plus a subset of the C2/curated gene sets collection. For the latter, gene sets from the following contributors were used: Biocarta, GO, GenMAPP, KEGG, Sigma-Aldrich, Signaling transduction KE and Signaling Alliance. GSEA was run with default settings (1000 gene set permutations). Gene sets with 5% FDR were considered significant in accordance with GSEA recommendations (GSEA user guide; http://www.broad.mit.edu/gsea/doc/GSEAUserGuideFrame.html).

## Results

### Experimental Design

**Figure 1:**
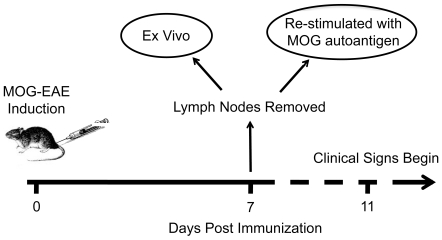
Experimental design. DA (n = 4) and PVG (n = 4) female rats were immunized with MOG (day 0) to induce EAE. At day 7, before clinical symptoms, lymph nodes were extracted and either directly harvested (*ex vivo*) or stimulated with autoantigen (MOG re-stimulated). cRNA was prepared and gene expression tested using 16 Affymetrix Rat Exon 1.0 ST Arrays.

**Figure 2:**
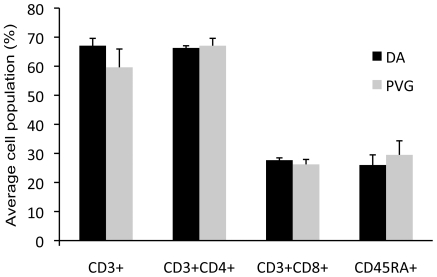
Cell types from lymph nodes 7 days after immunization. DA (n = 4) and PVG (n = 4) have equivalent proportions of CD3^+^ T cells as well as the CD4^+^ and CD8^+^ subsets, in addition to equal CD45RA^+^ B cells. Error bars represent standard deviation.

DA and PVG rats were immunized with MOG to induce EAE. MHC-identical rat strains were used in order to investigate the role of non-MHC influences in alternative splicing and differential expression during EAE. After 7 days of the autoimmune response inguinal lymph nodes were extracted from 4 animals of each strain. Lymph node cells were divided into two conditions: (i) direct *ex vivo* and (ii) MOG re-stimulation (Figure 1). No differences between strains in lymphocyte types (CD3^+^, CD4^+^, CD8^+^ T cells or CD45RA^+^ B cells) were detected using flow cytometry (Figure 2). The day 7 *ex vivo* cells represent an early disease stage when the immune response towards antigen has been mounted in the draining lymph node, but before leukocyte infiltration into the CNS and clinical signs are evident. MOG re-stimulation mimics the encounter between autoreactive cells and their target in the CNS, where effector molecules such as interferon-γ (IFN-γ), interleukin-17 (IL-17) and IL-22 become up-regulated [7].

### Alternative Splicing between Susceptible and Resistant Strains in EAE

**Figure 3:**
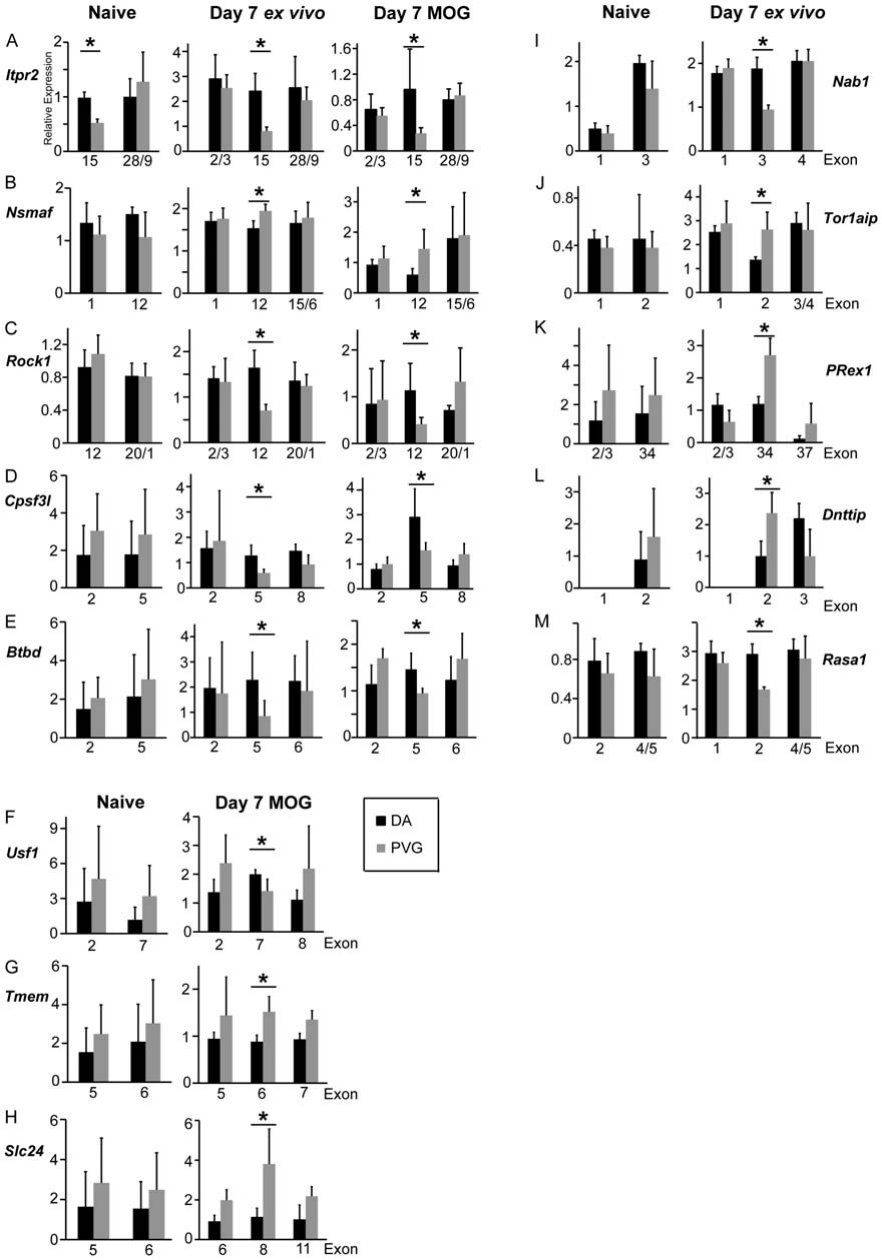
Quantitative PCR confirmation of alternative splicing identified constitutive and EAE-specific isoforms. Relative expression compared to *Hprt* from DA (n = 4) and PVG (n = 4) naïve (A–E and I–M), day 7 post-immunization (A–E and I–M) and MOG re-stimulated (A–H) conditions for selected target exons of alternatively spliced candidate genes. *Itpr2* had constitutive isoforms while all other genes displayed EAE-specific regulation. *Dnttip* exon 1 was not determined due to technical issues. * p<0.05 using a Mann-Whitney non-parametric test. Error bars represent standard deviation.

We investigated the role that alternative splicing plays in EAE induction and susceptibility using three parallel analytic approaches: MiDAS, Rank Product of the Splice Index and an Alternative Splicing ANOVA, followed by visual filtering. We first compared exon expression from the DA vs. PVG day 7 *ex vivo* conditions. 11 candidate genes for alternative splicing were identified (Table 1; Figure S1). We used the Ensembl (http://www.ensembl.org) and Interpro (http://www.ebi.ac.uk/interpro) databases to evaluate if the alternate isoforms may lack important protein functions. The isoforms for 9 of the 11 genes had putative altered protein function. The alternative splicing analysis was confirmed and quantified between strains using qPCR with primers designed for specific isoforms of each candidate (Figure 3). *Itpr2* isoforms were constitutively expressed for each strain in naïve and immunized lymph node cells (Figure 3A) while all other candidates except *Ddx19a* displayed EAE-specific regulation, as naïve lymph node cells did not display the same isoform expression patterns.

**Table 1:** Alternatively spliced genes between DA and PVG rats in day 7 *ex vivo* lymph nodes.

Ensembl ID	Transcript ID	Gene Symbol	p-value	Differential Exon	Reduced Strain	Interpro Domain	Domain Function
T00000032767	7271202	Itpr2	7.01E-26	15	PVG	-	No specific domain
T00000017813	7354298	Nab1	4.14E-17	3	PVG	IPR006986	Nab1, negative regulation of transcription
T00000014289	7283429	Nsmaf	2.99E-13	12	DA	IPR000409	Beige/Beach, unknown
T00000005280	7112632	Tor1aip1	9.72E-12	2	DA	IPR008662	Lamina-assoc polypeptide 1C, membrane attachment
T00000009517	7248004	P-Rex1	1.64E-11	34	DA	IPR001849	Pleckstrin homology, intracellular signaling
T00000040922	7173094	Rock1	5.90E-10	12	PVG	IPR011009	Protein kinase-like, phosphotransfer
T00000036931	7198657	Dnttip2	1.99E-09	2	DA	-	Coil, coil domain
T00000026725	7282342	Cpsf3l	2.00E-09	5	PVG	IPR001279	Beta-lactamase-like, hydrolase
T00000059034	7185469	Ddx19a	1.51E-07	10	DA	-	No specific domain
T00000046251	7201908	Rasa1	3.17E-07	2 and 3	PVG	IPR001936	Ras GTPase-activating protein, signaling
T00000019338	7056128	Btbd10	2.34E-06	5	PVG	IPR003131	Potassium channel, voltage dependent

Differential Exon - alternatively spliced between strains; Reduced Strain - lower expression of indicated exon;

Interpro Domain - protein domain encoded in alternatively spliced exon.

We next tested if alternative splicing was involved after MOG re-stimulation, which mimics the autoantigen re-encounter that occurs when autoreactive cells migrate to the CNS. We compared DA vs. PVG MOG re-stimulated exon expression and identified 9 alternatively spliced genes; 3 of which (*Usf1*, *Tmem41B* and *Slc24a6*) were MOG-re-stimulation specific (Table 2; Figure S2). We confirmed the Affymetrix results using qPCR for each of the candidate genes (Figure 3). The isoforms for 6 of the 9 genes had putative altered protein function. We then compared the two strains individually (i.e. *ex vivo* vs. MOG re-stimulated for DA and PVG separately). We identified a single alternatively spliced gene between DA conditions, the acetylcholine receptor ε subunit (*Chrne*; p = 7.77E-11; Figure S3A). The last 2 exons at the 3′ end were reduced after re-stimulation and would result in the loss of the signaling peptide. A single alternatively spliced gene was identified when comparing PVG conditions, the cAMP response element modulator (*Crem*; p = 1.95E-06; Figure S3B). The first 2 exons at the 5′ end were reduced after re-stimulation although no specific domain is associated with this portion of the gene.

**Table 2:** Alternatively spliced genes between DA and PVG cells after MOG re-stimulation.

Ensembl ID	Transcript ID	Gene Symbol	p-value	Differential Exon	Reduced Strain	Interpro Domain	Domain Function
T00000032767	7271202	Itpr2	6.41E-42	15	PVG	-	No specific domain
T00000014289	7283429	Nsmaf	1.29E-18	12	DA	IPR000409	Beige/Beach, unknown
T00000005811	7107800	Usf1	7.80E-13	7	PVG	IPR001092	Basic Helix-Loop-Helix, DNA binding + transcription
T00000040922	7173094	Rock1	6.34E-12	12	PVG	IPR011009	Protein kinase-like, phosphotransfer
T00000026725	7282342	Cpsf3l	5.18E-11	5	PVG	IPR001279	Beta-lactamase-like, hydrolase
T00000016224	7055841	Tmem41b	1.96E-09	6	DA	-	Transmembrane Helix
T00000059034	7185469	Ddx19a	2.79E-08	10	DA	-	No specific domain
T00000019338	7056128	Btbd10	3.32E-07	5	PVG	IPR003131	Potassium channel, voltage dependent
T00000001868	7102483	Slc24a6	4.44E-05	8	DA	-	No specific domain

Differential Exon - alternatively spliced between strains; Reduced Strain - lower expression of indicated exon;

Interpro Domain - protein domain encoded in alternatively spliced exon.

Mismatches between a microarray probe and its target sequence affect hybridization that cause erroneous probe signal estimates [28]. To determine if genetic differences (single nucleotide polymorphisms for instance) have affected our alternative splicing detection rates, we sequenced the exons of 4 genes that were alternatively spliced in both *ex vivo* and re-stimulated conditions. No genetic variation was discovered in *Itpr2*, *Nsmaf* and *Cpsf3l* (data not included). However, a SNP was found in probe-set 5733439 of *Ddx19a* exon 10, which could affect intensity values (Figure S4A). Accordingly, we could not reproduce the *Ddx19a* exon 10 Affymetrix data using qPCR (Figure S4B).

### Differential Expression and Pathway Regulation between Susceptible and Resistant Strains

To assess EAE-relevant genes we compared day 7 *ex vivo* expression between EAE-susceptible DA and EAE-resistant PVG rats. At a 5% FDR cut-off margin 206 transcripts were differentially expressed (Table S2). DAVID clustering analysis identified five biological pathways that were significantly affected during early EAE stages: glycosylation, apoptosis, synaptic transmission, extracellular structure organization and cellular differentiation (Table 3). GSEA identified a single functional gene set, sulfotransferase activity, which was enriched in PVG *ex vivo* lymphocytes after correcting for multiple hypothesis testing (Table 4). Interestingly, two additional gene sets that fell just short of the significance threshold were also positively associated with PVG *ex vivo* lymphocytes. They contained genes involved in neuroactive ligand-receptor interaction and substrate specific channel activity.

**Table 3:** DAVID enriched GO terms of differentially expressed genes between DA and PVG for day 7 *ex vivo* lymph nodes.

Category	Representative GO Term	Genes	Count/List	Pop Hits/Total	Fold Enrichment	p-value
GO∶BP	GO∶0030154∼cell differentiation	Nuak2, Agrn, Cd5, Nrp2, App, Il2ra, Actn1, Cstb, F2r, Rps6ka2, Igf1r, Capn5, Apbb1, Anxa1, Zfp37,	18/83	1584/12762	1.75	2.21E-02
GO∶BP	GO∶0006493∼protein amino acid O-linked glycosylation	Pomgnt1, Galnt11, Galnt10,	3/83	17/12762	27.13	5.21E-03
GO∶BP	GO∶0042981∼regulation of apoptosis	Nuak2, Cd5, App, Igf1r, Il2ra, Actn1, Anxa1, F2r, Cstb,	9/83	525/12762	2.64	1.93E-02
GO∶BP	GO∶0007268∼synaptic transmission	Gnai1, Vdac3, Agrn, Sv2b, App, Nsg1, Stx3,	8/83	446/12762	2.76	3.93E-02
GO∶BP	GO∶0043062∼extracellular structure organization and biogenesis	Agrn, App, Apbb1, F2r,	4/83	98/12762	6.28	2.51E-02

GO∶BP - Gene Ontology, Biological Process; p value - modified Fischer Exact Test.

**Table 4:** GSEA enriched gene sets between DA and PVG.

NAME	Top 10 genes	Source	SIZE	NOM p-val	FDR q-val
***DA vs. PVG day 7 ex vivo significant pathways***					
SULFOTRANSFERASE_ACTIVITY	Chst9, Chst7, Chst10, Chst13, Tpst1, Hs3st5, Chst3, Chst5, Chst8, Chst4	GO	20	0	0.03662996
HSA04080_NEUROACTIVE_LIGAND_RECEPTOR_INTERACTION	Ptger3, P2ry14, Gzma, Uts2R, Prl, Mtnr1a, Ghsr, P2ry4, Grin2B, Htr1B	KEGG	205	0	0.0560383
SUBSTRATE_SPECIFIC_CHANNEL_ACTIVITY	Kcna1, Cacng5, Kcnj15, Kcna4, Nmur2, Kcnb2, Kcne2, P2rx3, Kcnk4, Scn2b	GO	131	0	0.07680687
***DA vs. PVG MOG restimulation significant pathways***					
VOLTAGE_GATED_POTASSIUM_CHANNEL_ACTIVITY	Kcnj4, Kcnj3, Kcnj1, Kcnip2, Kcna5, Kcnh1, Kcnj10, Kcna2, Kcnc1, Kcnj15	GO	31	0	0.014385469
FEEDING_BEHAVIOR	Mc4r, Npy, Ghrl, Htr2c, Lep, Npw, Mchr1, Calr2, Hcrtr2, Ppyr1	GO	21	0	0.015389259
POTASSIUM_ION_TRANSPORT	Kcnj4, Kcnj2, kcnj3, Kcnj1, Kcnj11, Kcnip2, Kcna5, Kcnh1, Kcnj10, Kcnf1	GO	49	0	0.027649486
POTASSIUM_CHANNEL_ACTIVITY	Kcnj4, Kcnj3, Kcnj1, Kcnip2, Kcna5, Kcnh1, Kcnj10, Kcna2, Kcnc1, Kcnj15	GO	41	0	0.02914241
HORMONE_ACTIVITY	Gh1, Npy, Ghrl, Igf1, Stc1, Cga, Nppb, Calcb, Chgb, Pnoc	GO	36	0	0.029265244
GPCRDB_CLASS_A_RHODOPSIN_LIKE	Nmur1, Gpr85, Chrm5, Gpr83, Drd2, Mc4r, Fpr1, Gpr37, P2ry1, Grpr	GenMAPP	133	0	0.032977927
RESPONSE_TO_NUTRIENT_LEVELS	Nuak2, Npy, Ghrl, Lep, Stc1, Sst, Cdkn2b, Alb	GO	26	0.001677852	0.034934502
INTERMEDIATE_FILAMENT_CYTOSKELETON	Gfap, Drd2, Nefl, Krt31, Pkp1, Upp2, Krt2	GO	18	0.001658375	0.036004912
VOLTAGE_GATED_POTASSIUM_CHANNEL_COMPLEX	Kcnj4, Kcnj3, Kcnj1, Kcna5, Kcnh1, Kcna2, Kcnc1, Kcnj8, Kcns1, Kcnn3	GO	35	0	0.03604953
RESPONSE_TO_EXTRACELLULAR_STIMULUS	Nuak2, Npy, Ghrl, Lep, Stc1, Sst, Cdkn2b, Alb	GO	28	0.001712329	0.03640272
STRUCTURAL_CONSTITUENT_OF_CYTOSKELETON	Actl7a, Gfap, Nefl, Krt31, Sorbs3, Ppl, Des, Actl7b, Mapt, Krt2	GO	42	0	0.036414582
INTERMEDIATE_FILAMENT	Gfap, Drd2, Nefl, Krt31, Pkp1, Upp2, Krt2	GO	18	0.00172117	0.036638036
HSA04080_NEUROACTIVE_LIGAND_RECEPTOR_INTERACTION	Nmur1, Prss1, Npffr2, Chrm5, Gpr83, Gh1, Taar2, Drd2, Mc4R, Fpr1	KEGG	205	0	0.037446167
SYSTEM_PROCESS	Nmur1, Omp, Six3, Gja3, Sspn, Pmp22, Drd2, Npy, Nrl, Kcnj1	GO	441	0	0.038417198
HSA01430_CELL_COMMUNICATION	Chad, Gja3, Krt35, Krt34, Ibsp, Krt25, Krt31, Nes, Krt86, Krt40	KEGG	96	0	0.038440228
SYNAPTIC_TRANSMISSION	Omp, Pmp22, Drd2, Npy, Ghrl, Kcnip1, Htr2c, Pcdhb13, Pcdhb6, Htr1b	GO	151	0	0.038810864
NEUROLOGICAL_SYSTEM_PROCESS	Nmur1, Omp, Six3, Gja3, Pmp22, Drd2, Npy, Nrl, Ghrl, Kcnip1	GO	290	0	0.039604984
CARBOHYDRATE_TRANSPORT	Gh1, Ppbp, Ednra, Slc2a5	GO	15	0.001675042	0.046175405

Top 10 genes – top 10 ranking genes belonging to a gene set; source – source for the gene set; size – number of genes in a gene set; NOM p-val – nominal p value; FDR q-val – false discovery rate.

*In vitro* MOG re-stimulation of autoreactive cells from peripheral lymphoid tissue replicates the pathogenic reaction occurring in the CNS of affected rats and results in the up-regulation of disease-driving effector molecules. Comparing the two strains for expression differences after MOG re-stimulation, 144 genes were significant using 5% FDR (Table S3). DAVID clustering analysis identified 8 biological pathways that were regulated between the two strains upon MOG re-stimulation, including T cell activation, apoptosis and transport (Table 5). In addition, GSEA analysis highlighted that PVG cells up-regulated of a range of potassium channel genes (Table 4).

**Table 5:** DAVID enriched GO terms of differentially expressed genes between DA and PVG for MOG re-stimulated cells.

Category	Representative GO Term	First 10 Genes	Count/List	Pop Hits/Total	Fold Enrichment	p-value
GO∶BP	GO∶0051234∼establishment of localization	Drd2, Arfgap1, Camk2d, Atp10a, Cxcr4, P2rx4, Trpm6, Tpo1, Slc27a1, Dirc2	25/76	2420/12762	1.73	4.77E-03
GO∶BP	GO∶0012501∼programmed cell death	Cd2, Litaf, Nuak2, Cd5, App, Fgf2, Igf1r, Il2ra, Cxcr4, F2r	11/76	690/12762	2.68	6.88E-03
GO∶BP	GO∶0006464∼protein modification process	Pigq, Nuak2, Pomgnt1, Art2b, Camk2d, St8sia1, Cry2, Uhrf1, Insr, Atg7	19/76	1403/12762	2.27	1.03E-03
GO∶BP	GO∶0030154∼cell differentiation	Litaf, Nuak2, Cd5, Nrp2, App, Il2ra, Sort1, Cxcr4, F2r, Cd2	16/76	1584/12762	1.70	4.05E-02
GO∶BP	GO∶0046649∼lymphocyte activation	Cd2, Cd5, Nbn, Il2ra, Cxcr4	4/76	105/12762	6.40	2.38E-02
GO∶BP	GO∶0008152∼metabolic process	Drd2, Gfpt2, Cdo1, St8sia1, Cybrd1, Lmo7, Chst10, Trpm6, Capn5, Khdrbs3	51/76	7051/12642	1.21	2.91E-02
GO∶BP	GO∶0000902∼cell morphogenesis	Nrp2, App, Fgf2, Igf1r, Camk2d, Socs2, Atp10a, Cxcr4	8/76	468/127642	2.87	1.98E-02
GO∶BP	GO∶0042110∼T cell activation	Cd2, Cd5, Il2ra, Cxcr4	4/76	105/12762	6.40	2.38E-02
GO∶BP	GO∶0048856∼anatomical structure development	Drd2, Nrp2, App, Camk2d, Il2ra, Csrp1, Sort1, Atp10a, Hsd11b1, Insr	19/76	1988/12762	1.60	3.69E-02

GO∶BP - Gene Ontology, Biological Process; p value - modified Fischer Exact Test.

We next examined genes that were differentially expressed upon autoantigen re-stimulation for each strain separately. Comparing DA day 7 *ex vivo* versus DA MOG re-stimulated conditions, 903 genes showed differential expression using 5% FDR (Table S4), while the same comparison for PVG identified 1356 differentially expressed genes (Table S5). DAVID clustering analyses revealed that similar GO pathways were regulated in both strains. Both DA and PVG respond to stimulus by regulating metabolism, transport, kinase activity, transcription and apoptosis, which ultimately control the immune response (Tables S6 and S7). Although similar pathways were regulated in both strains, some of the genes associated with these clusters were strain specific. For example, sterol metabolism was highly enriched when comparing day 7 *ex vivo* versus MOG re-stimulated conditions for each strain separately, with 16 common associated genes. However, *Mvd*, *Oprs1* and *Hmgcs1* were identified only for DA, while *Osbpl5* and *Ebp* were differentially expressed only for PVG.

### Candidate Genes for EAE QTLs

**Figure 4:**
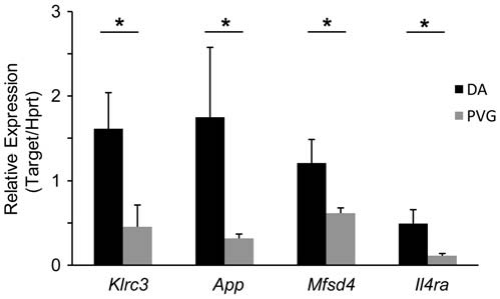
Quantitative PCR confirmation of candidate genes for EAE QTLs. Relative expression compared to *Hprt* of *Klrc3*, *App, Mfsd4* and *Il4ra* from DA (n = 4) and PVG (n = 4) female rat lymph nodes, 7 days post-immunization. DA lymph nodes had higher expression of all 4 targets, confirming the Affymetrix data. * p<0.05 using a Mann-Whitney non-parametric test. Error bars represent standard deviation.

Several of the differentially expressed genes and an alternatively spliced gene identified in this study overlap with known QTLs between DA and PVG (Table 6). We confirmed the Affymetrix data using qPCR for 4 of these targets: *Il4ra*, *Klrc3*, *App* and *Mfsd4* (Figure 4). These genes can be considered as candidate genes governing EAE susceptibility in these regions.

**Table 6:** Candidate genes for EAE quantitative trait loci.

QTL	Chromosome	Position (Mb)	Candidate Genes	Related Publication
*“Eae31”*	1	183.9–193.1	Il4ra, Acadsb	
*Unnamed*	1	234.5–268	Lipa, Ifit1, Pdlim1, RGD1303232	
*'Eae24-27''*	4	44–116.6	Scrn1, LOC681217, Gimap4, Reg3g, *Tspan33, Chn2*	Becanovic et al., 2003
*Eae20*	4	157–160.5	*Slc6a12*	Jagodic et al., 2005
*Eae22*	4	∼167	Klrc3	Jagodic et al., 2005
*Unnamed*	*5*	*125.6–173*	Dmrtb1, Spata6, Pomgnt1, Csf3r, Agrn, *Acot7,* **Cpsf3l**	
*'Eae16''*	8	81.4–102.9	Lrrc, *Plscr2*	
*Eae18a*	10	55.8–62.7	Plscr3, Zmynd15, Eno3, *Cldn7, Kif1c*	Jagodic et al., 2004
*Unnamed*	*11*	*20–70*	App, Mx1, Retnlg, Cd200r1, Parp9, Dirc2	
*'Eae17''*	13	39.6–55.4	Mfsd4, Nuak2, Pik3c2b, Chi2l1, Cfh, *Cxcr4*	
*Unnamed*	*14*	*0–26.4*	Naaa, *Slc4a4*	
*Eae19*	15	83.3–97.7	Lmo7, *Spry2*	Sheng et al., 2005
*'Eae23''*	17	36–66	Pols	

QTLs in quotations or unnamed are based on personal communication; Genes - differentially expressed between DA and PVG in day 7 *ex vivo* lymph node cells; *Genes -* differentially expressed in MOG re-stimulated cells; Genes - differentially expressed in both *ex vivo* and re-stimulated conditions; **Genes** - alternatively spliced.

## Discussion

We investigated the induction phase and autoreactive cell effector functions during EAE between susceptible DA and resistant PVG inbred rat strains using whole genome expression profiling of peripheral lymphatic tissue. Our aim was to assess the role of alternative splicing and to identify genes that were responsible for susceptibility to the disease. We hypothesized that alternative splicing, a mechanism that widely occurs between tissues and across time [29], is an important mechanism involved in disease regulation. Additionally, we sought to identify candidate genes and pathways responsible for genetically-determined EAE susceptibility, some of which may also regulate the human disease, MS.

Our study identified a total of 13 alternative spliced genes between susceptible and resistant strains. This result is likely an underestimation of the true event number with many false-negatives (Type II error) due to at least two reasons. Firstly, we have used a mixed population of lymph node cells, which could mask the contribution of individual cell type specific alternative spicing. Secondly, we employed several methods to increase stringency, as most of the alternative splicing detection methods suffer from high false positive detection rates [30]. We observed genes that were differentially spliced between EAE-susceptible and -resistant rat strains during disease induction and effector phases. When examining the missing exons we determined that the isoforms for many genes are missing integral protein domains, thus providing support that these differences functionally contribute to disease susceptibility. The spliced genes are involved in several general signaling and transcriptional regulatory mechanisms that were identified in this study. *Rasa1* and *Rock1* alternative splicing was EAE-specific and could control cell motility, proliferation and differentiation through Ras and Rho GTPases respectively [31], [32]. Additionally, *Nab1*, *Cpsf3l*, *Btbd10* and *Usf1* all modulate transcription [33], [34], [35], [36]. However, further investigation is required to determine the precise effect these different isoforms have during EAE induction. Interestingly, 5 of the 10 genes identified (this excludes *Ddx19a*, which could not be reproduced likely due to a SNP affecting hybridization) as alternatively spliced in day 7 lymph nodes were also spliced after MOG re-stimulation. This result indicates that these isoforms are constitutive during disease and may govern disease-driving mechanisms.

Expressional regulation is an important mechanism modulating biological processes. Many genes are differentially expressed during disease, providing markers of disease progression and indications of pathways and mechanisms that are deregulated. We identified 206 genes that were differentially expressed between the two stains in day 7 post-immunization lymph nodes. Of those genes, *Cd5, Il18r1* and *Il2ra* are known encephalomyelitis candidates [7], [37], [38]. All three are strongly up-regulated in DA lymph nodes. Disease-driving regulated genes in EAE are often related to the immune system and govern processes including antigen processing, transcription, cytokines and cellular attraction, adhesion, division and death. Our study supports this body of evidence but also extends the findings to propose new candidates governing autoimmunity that are implicated in glycosylation (*Pomgnt1*, *Galnt11* and *Galnt10*). Our study focused on the periphery where protein glycosylation may affect receptor signaling and thereby alter disease susceptibility [39]. Additionally, pathogenic glycosylated antigens are generated during EAE and antibodies against these targets perturb disease [40].

After MOG re-stimulation we identified 144 genes that were differentially expressed between the two strains. One the genes strongly up-regulated in DA lymph nodes was *Havcr1/Tim1*, which was also increased in cerebrospinal fluid mononuclear cells of patients with MS [41]. GSEA analysis identified up-regulation of several members of the inward rectifier potassium channel family, including *Kcnj1* and *Kcnj4*, in PVG cells, suggesting that these channels may play a protective role in EAE. *Kcnj4* (or *Kir2.3*) has recently been linked with protection against neurodegeneration in a cell model of Parkinson's disease [42], although its role in the immune system is currently unknown. There is strong interest in targeting potassium channels for MS therapy and several clinical trials related to the therapeutic targeting of voltage-gated K^+^ (K_v_) channels in MS are ongoing [43]. These therapies will likely not only target nervous tissue and conductance, but also lymphocyte proliferation [44], [45]. Additionally, K_v_ channels regulate immune synapses in the periphery where antigen presentation occurs and lymphocyte function is determined [46].

It should be noted that stringent criteria were used for all our analysis. We chose this approach to simplify interpretation of the data, reduce the occurrence of false positives and provide strong novel EAE susceptibility candidates. Because of the stringent criteria, we find that only a portion of the genes from our previous study [7], which utilized qPCR methods, pass the 5% FDR cut-off, including *Il18r1*, *Il2ra* and *FoxP3*. However, genes that do not pass a FDR of 5% in this study, including *Il17f*, *Il7r*, *Stat4* and *Tnf*, are consistently differentially expressed at a lower threshold (p<0.05). When examining known MS candidates we found that both *ll7r* and *Il2ra* were confirmed as being differentially expressed between DA and PVG in day 7 *ex vivo* lymph nodes (p = 0.04 and p = 8×10^−5^, respectively). We also determined differential expression of *Evi5* (p = 0.03) in *ex vivo* cells and *Irf8*/*Icsbp1* (p = 0.02) following MOG re-stimulation [47], [48]. These results provide further evidence that MOG-induced EAE in DA rats is a relevant model for study of MS. However, not all MS candidate molecules are transcriptionally regulated at this time-point in this tissue. Candidate genes including *KIF1*β [49], *RPL5* [50], *IRF5* [47], *CD226* [51], *CD6* and *TNFRSF1A* [48] were equally expressed between susceptible and resistant strains.

Several studies have employed linkage analysis with QTL mapping to identify genomic regions regulating EAE in rats [52], [53], [54], [55], [56]. However, identification of the genetic polymorphisms responsible for disease susceptibility has been a slow process. We combined QTL mapping and expression profiling, a strategy that has been successful at identifying the molecular basis of a number of QTLs in rats [57], [58], [59], [60]. We have identified numerous differentially expressed and alternatively spliced genes overlapping previously described QTLs for EAE between DA and PVG and confirmed several of them using qPCR. One candidate from our study, *Cxcr4*, is involved in leukocyte trafficking and has previously been implicated in EAE [61]. Another candidate, *Klrc3*, encodes an activating natural killer (NK) cell receptor, which could potentially regulate NK cell involvement in EAE [62], [63]. We propose these and other genes as candidates in the respective genetic regions and believe that directed study will help identify pathways regulated during autoimmune reactions, giving further insight into human MS.

## Supporting Information

Figure S1 Eleven genes alternatively spliced between DA and PVG day 7 *ex vivo* lymph node cells, as presented from Partek's gene viewer (A-K). The average RMA normalized intensity values and standard error for each probe-set, are shown for EAE-susceptible DA (blue) and EAE-resistant PVG (red) rat strains. The Log2 intensity scale is shown on the right axis. Arrows designate alternatively spliced exons.
(1.72 MB DOC)

Figure S2 Nine genes alternatively spliced between DA and PVG MOG re-stimulated lymph node cells, as presented from Partek's gene viewer (A–I). The average RMA normalized intensity values and standard error for each probe-set are shown for EAE-susceptible DA (blue) and EAE-resistant PVG (red) rat strains. The Log2 intensity scale is shown on the right-had axis.Arrows designate alternatively spliced exons.
(1.05 MB DOC)

Figure S3 Alternative splicing genes from *ex vivo* vs. re-stimulated conditions, as represented from Partek's gene viewer. DA shows down-regulation of the 3′ end of Chrne after MOG re-stimulation (A). PVG shows down-regulation of the 5′ end of Crem after re-stimulation (B). The average RMA normalized intensity values and standard error for each probe-set are shown. Arrows designate alternatively spliced exons.
(0.54 MB DOC)

Figure S4 Ddx19a is an example of an alternatively spliced false-positive induced by a SNP. (A) Sequencing exon 10 of Ddx19a identified a SNP (arrows) in probe-set 5733439 between DA and PVG. (B) Relative expression compared to Hprt for Ddx19a determined no difference between DA (n = 4) and PVG (n = 4) for exon 10, the putative alternatively spliced exon. Error bars represent standard deviation.
(0.28 MB PDF)

Table S1 Primer sequences for SYBR quantitative real-time PCR targets.
(0.02 MB DOC)

Table S2 Differentially regulated transcripts between DA and PVG in day 7 *ex vivo* lymph nodes. 138 transcripts were upregulated and 68 downregulated using DA as reference at a 5% FDR cut-off margin.
(0.04 MB DOC)

Table S3 Differentially regulated transcripts between DA and PVG in MOG re-stimulated cells. 90 transcripts were upregulated and 54 downregulated using DA as reference at a 5% FDR cut-off margin.
(0.04 MB DOC)

Table S4 Differentially regulated transcripts comparing DA day 7 *ex vivo* and MOG re-stimulated conditions. 470 transcripts were upregulated and 433 downregulated using day 7 *ex vivo* as reference at a 5% FDR cut-off margin.
(0.13 MB DOC)

Table S5 Differentially regulated transcripts comparing PVG day 7 *ex vivo* and MOG re-stimulated conditions. 637 transcripts were upregulated and 719 downregulated using day 7 *ex vivo* as reference at a 5% FDR cut-off margin.
(0.18 MB DOC)

Table S6 DAVID functional clustering results of DA day 7 *ex vivo* and DA MOG re-stimulated conditions.
(0.02 MB DOC)

Table S7 DAVID functional clustering results of PVG day 7 *ex vivo* and PVG MOG re-stimulated conditions.
(0.03 MB DOC)
