# Supplementary figures and images for: Alternative Splicing and Transcriptome Profiling of Experimental Autoimmune Encephalomyelitis Using Genome-Wide Exon Arrays
Source: PLoS One. 2009 Nov 10;4(11):e7773. doi: 10.1371/journal.pone.0007773 (PMC2775719; doi:10.1371/journal.pone.0007773)

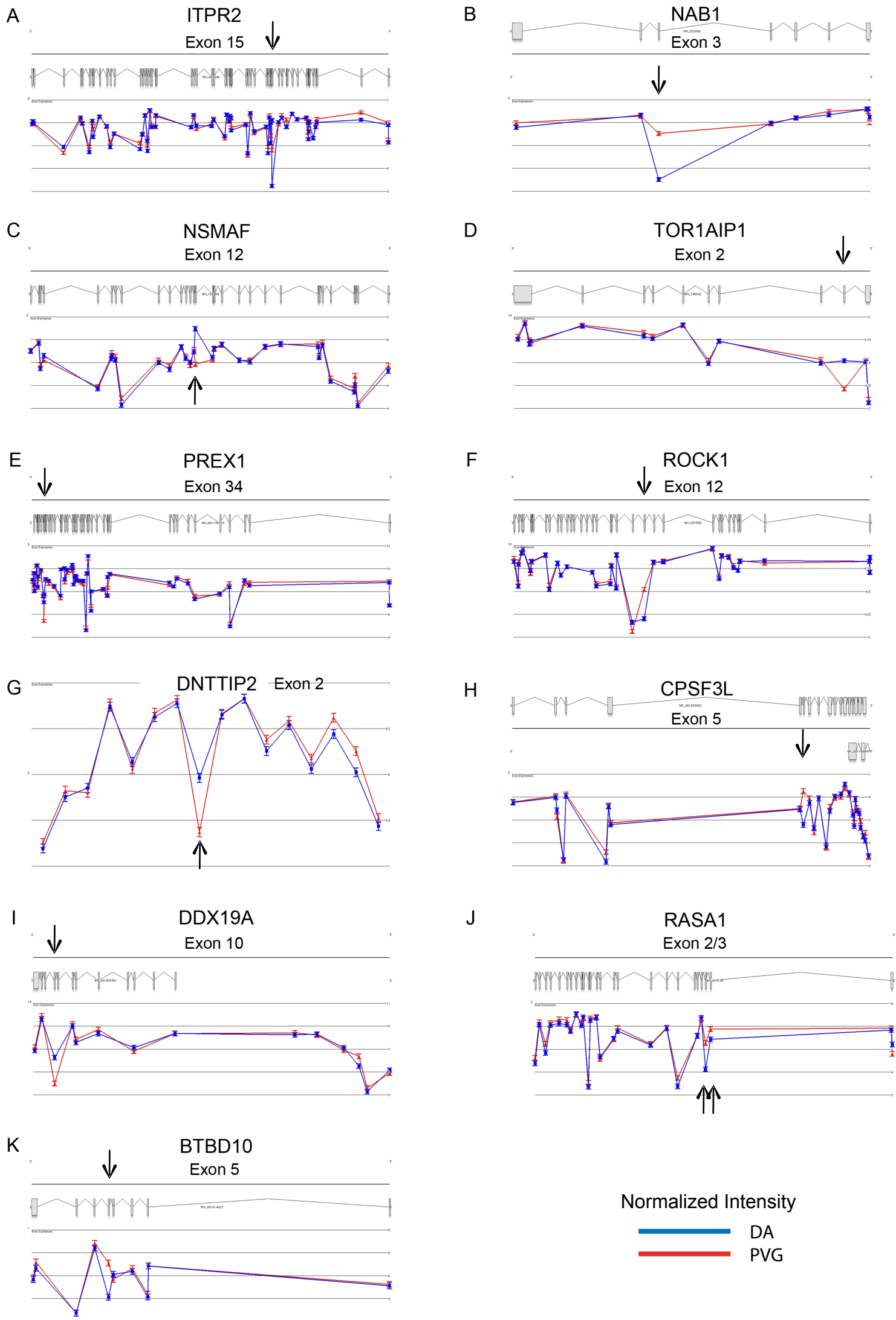

Supplement: Figure S1 — Eleven genes alternatively spliced between DA and PVG day 7 ex vivo lymph node cells, as presented from Partek's gene viewer (A-K). The average RMA normalized intensity values and standard error for each probe-set, are shown for EAE-susceptible DA (blue) and EAE-resistant PVG (red) rat strains. The Log2 intensity scale is shown on the right axis. Arrows designate alternatively spliced exons. (1.72 MB DOC) [file pone.0007773.s001.pdf]

A

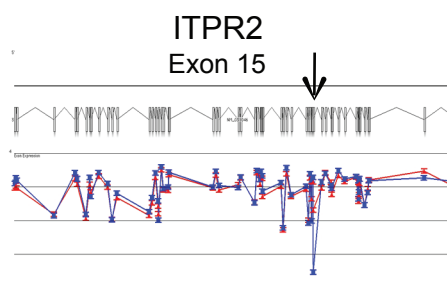

B

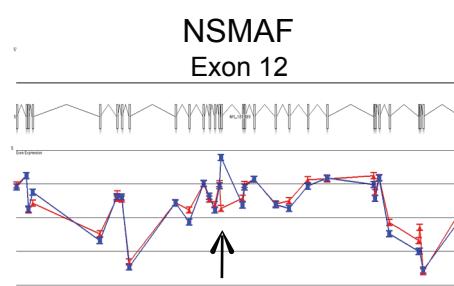

C

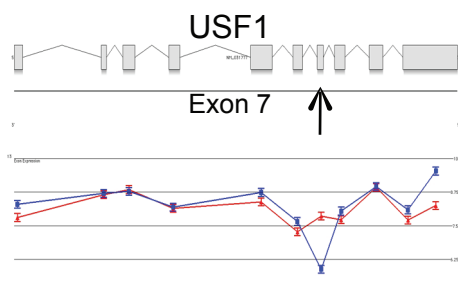

D

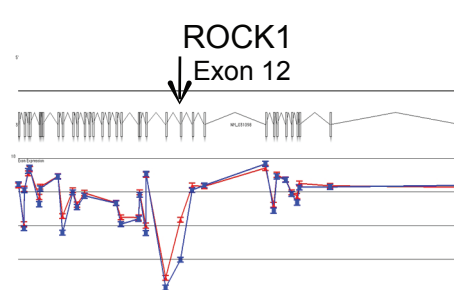

E

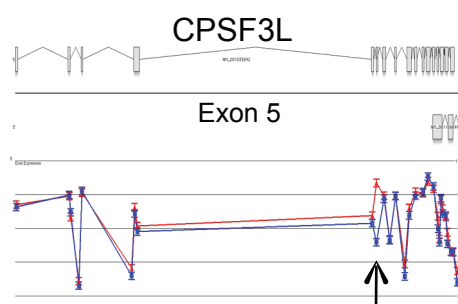

F

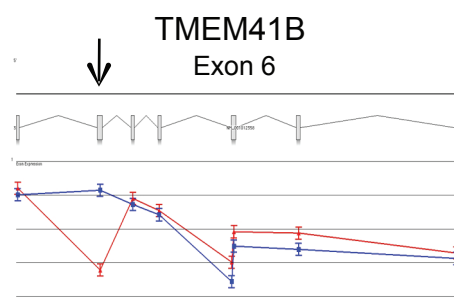

G

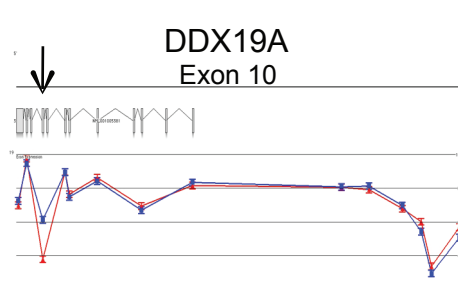

H

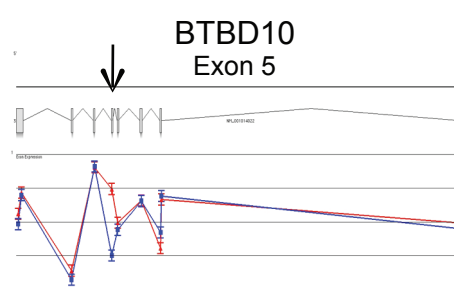

I

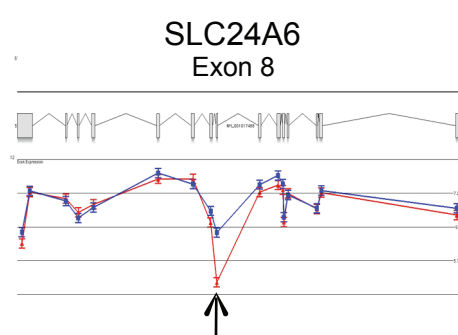

Normalized Intensity

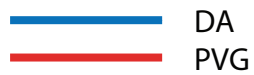

Supplement: Figure S2 — Nine genes alternatively spliced between DA and PVG MOG re-stimulated lymph node cells, as presented from Partek's gene viewer (A–I). The average RMA normalized intensity values and standard error for each probe-set are shown for EAE-susceptible DA (blue) and EAE-resistant PVG (red) rat strains. The Log2 intensity scale is shown on the right-had axis.Arrows designate alternatively spliced exons. (1.05 MB DOC) [file pone.0007773.s002.pdf]

A

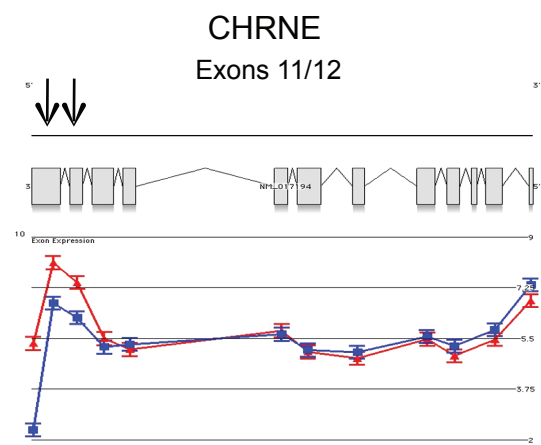

B

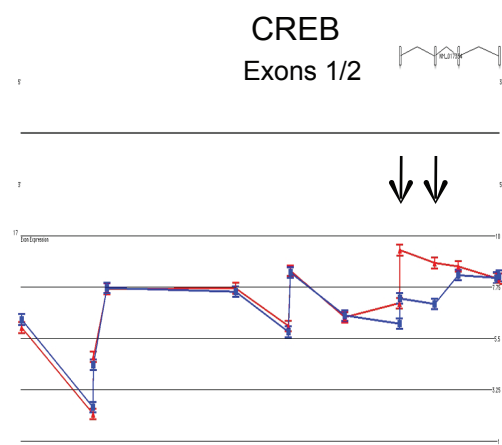

Normalized Intensity

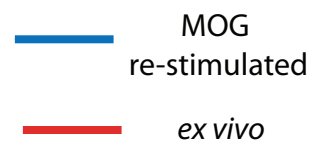

Supplement: Figure S3 — Alternative splicing genes from ex vivo vs. re-stimulated conditions, as represented from Partek's gene viewer. DA shows down-regulation of the 3′ end of Chrne after MOG re-stimulation (A). PVG shows down-regulation of the 5′ end of Crem after re-stimulation (B). The average RMA normalized intensity values and standard error for each probe-set are shown. Arrows designate alternatively spliced exons. (0.54 MB DOC) [file pone.0007773.s003.pdf]

A

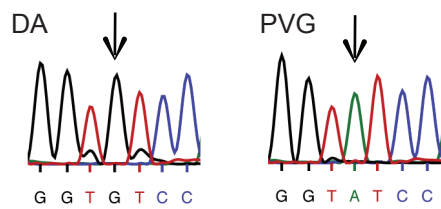

B

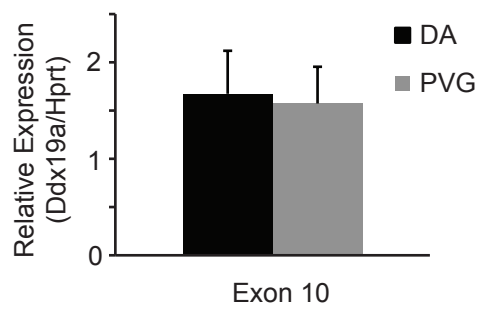

Supplement: Figure S4 — Ddx19a is an example of an alternatively spliced false-positive induced by a SNP. (A) Sequencing exon 10 of Ddx19a identified a SNP (arrows) in probe-set 5733439 between DA and PVG. (B) Relative expression compared to Hprt for Ddx19a determined no difference between DA (n = 4) and PVG (n = 4) for exon 10, the putative alternatively spliced exon. Error bars represent standard deviation. (0.28 MB PDF) [file pone.0007773.s004.pdf]
